# Arytenopexy with medialization thyroplasty and cricothyropexy in the treatment of unilateral vocal fold paralysis: A 15-year experience^☆^^☆☆^

**DOI:** 10.1016/j.bjorl.2025.101603

**Published:** 2025-04-24

**Authors:** Bernardo Scarioli Oliveira, Mauro Becker Martins Vieira, Flávia Amarante Cardoso, Lívia Bernardi Lopes, Marianna Novaes da Costa Avila, Kênia Rabelo Santana de Farias

**Affiliations:** Hospital Felicio Rocho, Departamento de Otorrinolaringologia, Belo Horizonte, MG, Brazil

**Keywords:** Vocal fold paralysis, Laryngoplasty, Vocal folds

## Abstract

**Objective:**

To evaluate our experience with the technique, functional results, and potential complications.

**Methods:**

A retrospective study was conducted with a sample of 29 patients submitted to this surgical procedure between 2000 and 2015. All procedures were performed under local anesthesia with sedation, and without intercurrences. Hospitalization time was 24 h on average.

**Results:**

None of the patients presented respiratory distress that required tracheostomy. Regarding vocal quality, the results were considered satisfactory or optimal, with high degree of patient satisfaction. With respect to dysphagia, all patients who needed enteral nutrition resumed exclusive peroral diet.

**Conclusion:**

Arytenopexy with medialization thyroplasty and cricothyropexy are procedures with low degree of complication that can be performed under local anesthesia with excellent functional results, even in patients with marked posterior glottic gap.

**Level of evidence:**

Level IV.

## Highlights

• Combined arytenopexy, thyroplasty, and cricothyropexy improved voice in unilateral vocal fold paralysis.
• All procedures were safely done under local anesthesia with sedation and no respiratory complications.
• Functional outcomes were satisfactory or optimal, even in cases with posterior glottic gap.
• Most patients resumed oral feeding post-op; aspiration and dysphonia were effectively corrected.
• Long-term complications were rare and did not significantly impact vocal outcomes.

## Introduction

Unilateral Vocal Fold Paralysis (UVFP) is not a rare finding in otorhinolaryngology. Its exact incidence has not been elucidated for several reasons. Many cases of UVPF are not diagnosed due to spontaneous recovery or contralateral vocal fold compensation.^1^

The etiology of this disease can be presented in four groups: neoplasia (caused by compression/infiltration of the vagus or recurrent laryngeal nerves); trauma (surgical or non-surgical); secondary to neurological disease; systemic and idiopathic.^2^ Therefore, before any treatment for functional disorders is proposed, careful assessment to identify the etiology of vocal fold paralysis should be conducted.

Therapeutic approaches include expectant observation, speech therapy, and different surgical techniques. Decision on treatment depends on the clinical condition of each patient. Some cases present little symptoms, requiring no specific treatment; whereas others, especially those of adduction paralysis, may present significant dysfunction of the larynx, with dysphonia, vocal fatigue, and gagging.^3^ Even in the latter cases, it is worth remembering that there may be recovery of vocal fold mobility or compensation from the opposite vocal fold with symptom improvement. This compensation mechanism can be optimized with the use of speech therapy. In situations where significant dysfunction persists, surgical treatment should be provided.

The specific scientific literature presents several techniques proposed for the improvement of these symptoms, with emphasis on the injection of substances in the vocal fold and the medialization surgeries standardized by Isshiki et al.^4^, ^5^ and Isshiki.^6^ Nevertheless, these techniques are often insufficient for cases that present significant posterior glottic gap with severe vocal quality impairment associated with constant gagging. Zeitels devised an adduction arytenopexy associated with vocal fold medialization and cricothyropexy to improve functional outcomes in these patients.^7^, ^8^, ^9^ As the otorhinolaryngology service where we practice presents a significant percentage of cases with these characteristics of posterior glottic gap, we began performing this surgical procedure. The purpose of this study is to present our experience with patient selection, methodology used, difficulties found, and results obtained.

## Methods

Inclusion criteria comprised patients undergoing arytenopexy with medialization thyroplasty and cricothyropexy between 2000 and 2015. Patients that presented Unilateral Vocal Fold Paralysis (UVFP) with marked posterior glottic gap, leading to symptoms of dysphagia and dysphonia, were referred to the procedure. A 6-month monitoring period was usually observed for UVFP reversal or compensation from the opposite vocal fold awaiting symptom improvement, except for cases of definite permanent etiology presenting severe aspiration symptoms with risk of malnutrition and pneumonia.

Prior to referral to the procedure, the patients underwent clinical evaluation to identify possible etiologies. This assessment was conducted individually and often included the following examinations: Computed Tomography (CT) of the neck to evaluate the vagus and recurrent laryngeal nerves, X-Ray of the chest, and rigid videolaryngoscopy.

Surgical procedures were performed under local anesthesia using 2% lidocaine, 1:100,000 epinephrine, and sedation. Flexible fibroscopy coupled with phonatory assessment were conducted during surgery to evaluate the intraoperative functional outcome. Unless contraindicated, the patients were given 0.2 mg/kg of dexamethasone associated with antibiotic prophylaxis 30 min before the procedure.

**Fig. 1:**
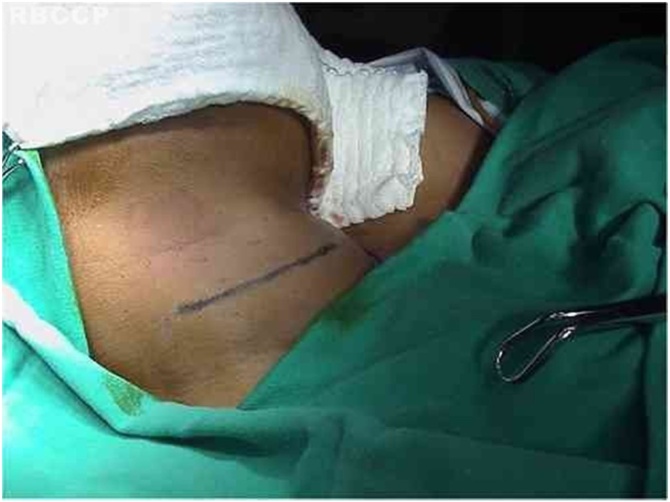
Transverse cervical incision, median, with ipsilateral extension.

**Fig. 2:**
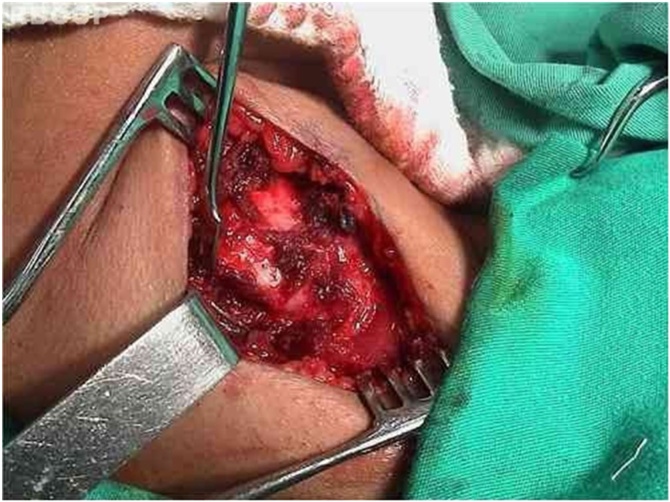
Section of the pre-thyroid muscles with exposure of the cricoid and thyroid cartilage.

**Fig. 3:**
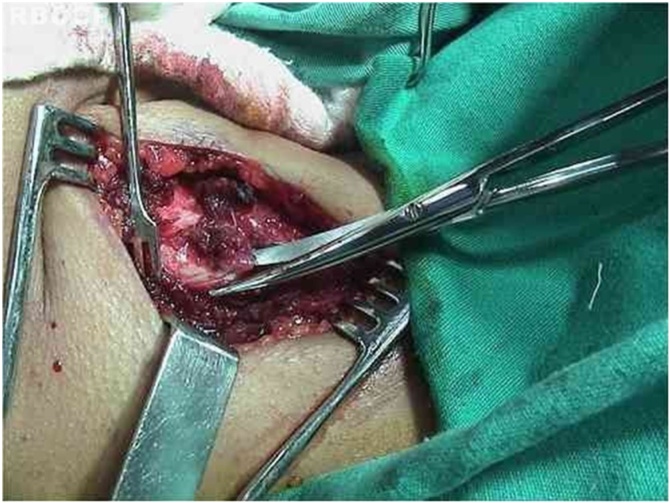
Disarticulation of the inferior cricothyroid joint.

**Fig. 4:**
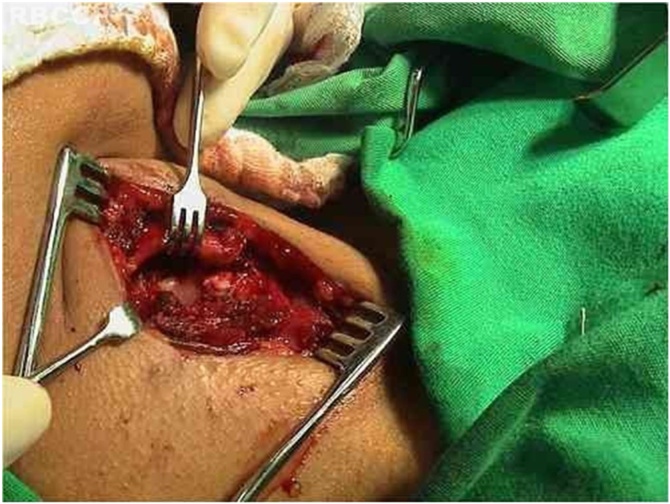
Anteromedial traction of the thyroid cartilage.

**Fig. 5:**
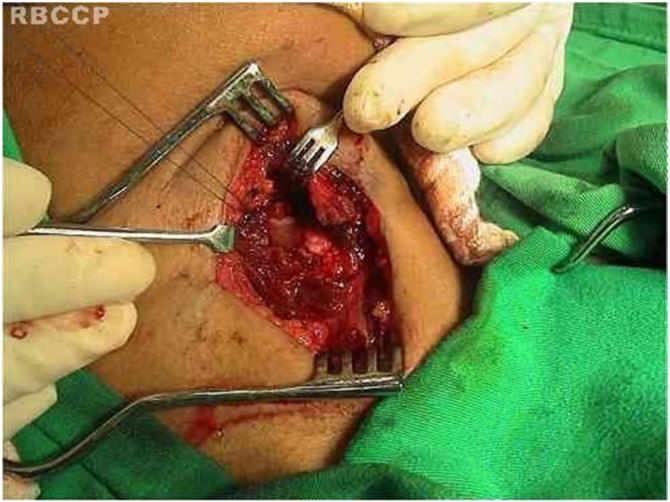
Arytenopexy leading to internal rotation and medialization of the arytenoid.

**Fig. 6:**
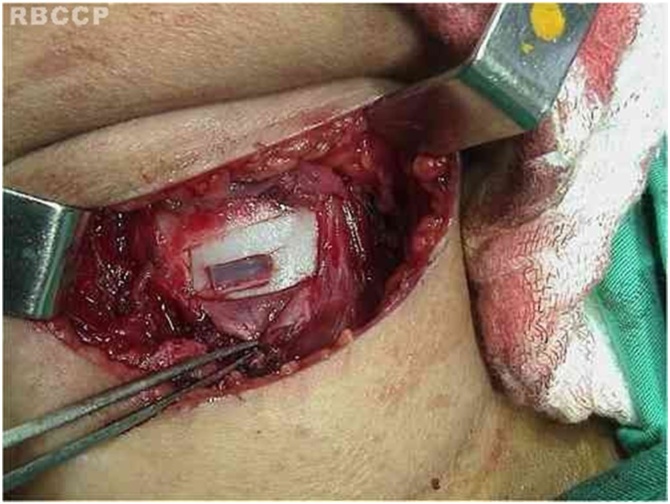
Type I thyroplasty.

**Fig. 7:**
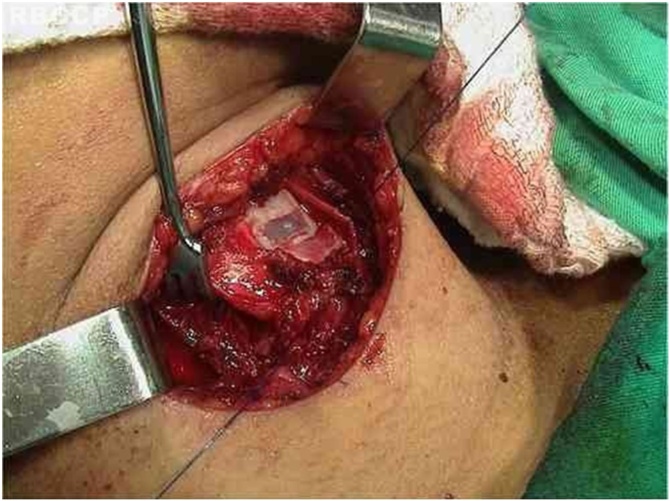
Suture of the inferior cornu of the thyroid cartilage in the anterior lamina of the cricoid cartilage.

The technique (Figs. 1‒7 ) consists of a transverse incision in the median cervical region with lateral extension to the paralyzed side at the inferior rim level of the thyroid cartilage, including skin, subcutaneous tissue, and platysma muscle. The incision edges are displaced in the upper and lower directions in a plane below the platysma, exposing from the superior rim of the thyroid cartilage to the inferior rim of the cricoid cartilage. The sternohyoid, sternothyroid and thyrohyoid muscle muscles are sectioned, providing a broad exposition of the ipsilateral ala of the thyroid cartilage.

A skin hook is placed lateral to the edge of the thyroid lamina and rotated anteromedially to define its edges and the lower cornu. An electrocautery knife is used to separate the inferior pharyngeal constrictor muscle from the thyroid lamina. The inferior cornu is identified and isolated so that the cricothyroid joint can be separated using scissors. Separation of the cricothyroid joint from the thyroid cartilage enables further anteromedial rotation of the thyroid lamina.

Dissection is performed under the superior rim of the cricoid cartilage until the superior rim of the arytenoid cartilage is encountered. During this procedure, the cricothyroid and lateral cricoarytenoid muscles are sectioned. The pyriform sinus mucosa is laterally folded.

With the cartilage exposed, the cricoarytenoid joint is opened with exposure of the articular facet of the cricoid cartilage. Prolene suture (5‒0) on a cutting needle is placed through the posterior plate of the cricoid just medial to the facet, and the needle is brought out through the medial aspect of the cricoarytenoid joint. After that, the needle is passed through the body of the arytenoid cartilage. The needle returns lateral to the vocal process of the arytenoid, is inserted in the inner portion of the superior rim of the cricoid, and is brought out through the posterior facet of the cricoid. This suture line is tensioned and a slipknot is placed, promoting medialization and internal rotation of the arytenoid cartilage with correction of the posterior glottic gap. The result is verified by videolaringoscopy and phonatory tasks. If the functional result is considered unsatisfactory, suture is redone.

Subsequently to adduction arytenopexy, type 1 thyroplasty is performed. A standard window is made in the thyroid lamina, preserving the inner layer perichondrium at the perimeter of the window. Material is placed to medialize the vocal fold. Silicone was used in the initial period of our practice; currently, expanded Polytetrafluoroethylene (ePTFE) is preferred. A thin sheet of ePTFE is introduced, and continuous perceptual evaluation of the patient’s voice is conducted. Material position is confirmed by visualization of the larynx through fibroscopy. Commonly, the vocal fold is left more medialized than necessary, as the intraoperative results will be modified after the local edema resulting from manipulation regresses. The material is supported by the external perichondrium and stabilized by suture in its original position.

Once the adduction arytenopexy and medialization thyroplasty are completed, a cricothyropexy, or cricothyroid subluxation, is performed to enhance vocal quality. Cricothyropexy is accomplished by placing a 2‒0 Prolene suture around the inferior cornu of the thyroid lamina, attaching it to the anterior portion of the cricoid. The suture is pulled taut, increasing the distance between the cricoid facet and the anterior commissure ligament. This suture increases the tension and length of the vocal fold on the paralyzed side, simulating cricothyroid muscle contraction.

The tension on this suture is adjusted by using a slipknot while the patient performs a number of phonatory tasks. The surgeon selects an appropriate tension for suture fixation, ensuring that the patient presents appropriate fundamental Frequency (Fo).

A Penrose drain is placed for 24 h. Patients are typically discharged on the first postoperative day.

## Results

The study sample was composed of 29 patients, with slight female predominance (15 cases) (Table 1). Mean age of participants was 58 years, ranging from 24 to 80 years. All patients tolerated the procedure under local anesthesia and sedation. No immediate complications were observed. There was no need for tracheostomy, and all patients were discharged on the first postoperative day.

**Table 1:** Descriptive data of the study sample.

Variables	Results
*Demographic data*	
Male gender	62 (77.5%)
Age (in years)	60.0 ± 10.3 (34‒87)
Subsite of primary lesion	
Oral tongue	45 (56.2%)
Floor of mouth	35 (43.8%)
Smoking	70 (87.5%)
Alcohol abuse	56 (70.0%)
*Anatomopathological data*	
Free resection margins	78 (97.5%)
Degree of differentiated	
Well differentiated	28 (35.0%)
Moderately differentiated	49 (61.3%)
Poor differentiated	3 (3.8%)
Perineural invasion	31 (38.8%)
Angiolymphatic invasion	13 (16.3%)
Tumor size^a^	23.2 ± 12.3 (1‒67) mm
Tumor thickness^a^	11.4 ± 8.5 (1‒39) mm
Depth of invasion^a^	11.3 ± 8.4 (1‒39) mm
Lymph node metastases	33 (41.3%)
Extracapsular spread (n = 25)	12 (48.0%)

a Mean ± Standard deviation (minimum‒maximum).

All individuals presented immediate functional results with significant improvement of the phonatory and swallowing functions. This improvement was reported as satisfactory or optimal by both the patients and the attending physicians.

In the long term, two patients presented late endolaryngeal extrusion of the material (one of silicone and one of ePTFE) used for medialization of the vocal fold. The materials were removed by direct laryngoscopy under microscopy and general anesthesia. Despite their removal, no significant worsening in vocal quality was observed.

Patients who had used nasoenteric tube or undergone gastrostomy preoperatively did not need them postoperatively (Table 2, Table 3, Table 4, Table 5).

**Table 2:** Comparison between the T clinical stages.

		T Classification (AJCC Cancer Staging System – 8^th^ edition)				
		T1	T2	T3	T4a	Total
T Classification (AJCC 7^th^ edition)	T1	5 (23.8%)	**11 (52.4%)**	**5 (23.8%)**	0 (0.0%)	21 (26.3%)
	T2	1 (1.7%)	**23 (39.0%)**	**35 (59.3%)**	0 (0.0%)	59 (73.8%)
	Total	4 (5.0%)	34 (42.5%)	42 (52.5%)	0 (0.0%)	80 (100.0%)

The main groups that received upstaging are shown in **bold**. p = 0.023 (Chi-Squared test).

**Table 3:** Comparison between the T clinical and pathological stages.

		T Classification (AJCC Cancer Staging System – 8^th^ edition)				
		T1	T2	T3	T4a	Total
T Classification (AJCC 8^th^ edition)	T1	4 (66.7%)	2 (33.3%)	0 (0.0%)	0 (0.0%)	6 (7.5%)
	T2	13 (38.2%)	12 (35.3%)	9 (26.5%)	0 (0.0%)	34 (42.5%)
	T3	1 (2.5%)	11 (27.5%)	**28 (70.0%)**	0 (0.0%)	40 (50%)
	Total	18 (22.5%)	25 (31.3%)	37 (46.3%)	0 (0.0%)	80 (100.0%)

The main groups that received upstaging are shown in **bold**. p = 0.023 (Chi-Squared test).

**Table 4:** Comparison between the N clinical stages.

		T Classification (AJCC Cancer Staging System – 8^th^ edition)							
		N0	N1	N2a	N2b	N2c	N3a	N3b	Total
T Classification (AJCC 8^th^ edition)	N0	46 (100%)	0 (0.0%)	0 (0.0%)	0 (0.0%)	0 (0.0%)	0 (0.0%)	0 (0.0%)	46 (57.5%)
	N1	0 (0.0%)	15 (88.2%)	**2 (11.8%)**	0 (0.0%)	0 (0.0%)	0 (0.0%)	0 (0.0%)	17 (21.3%)
	N2a	0 (0.0%)	0 (0.0%)	1 (100.0%)	0 (0.0%)	0 (0.0%)	0 (0.0%)	0 (0.0%)	1 (1.3%)
	N2b	0 (0.0%)	0 (0.0%)	0 (0.0%)	4 (40%)	0 (0.0%)	0 (0.0%)	**6 (60.0%)**	10 (12.5%)
	N2c	0 (0.0%)	0 (0.0%)	0 (0.0%)	0 (0.0%)	3 (50.0%)	0 (0.0%)	**3 (50.0%)**	6 (7.5%)
	Total	46 (57.5%)	15 (18.8%)	3 (3.8%)	4 (5.0%)	3 (3.8%)	0 (0.0%)	9 (11.3%)	80 (100%)

The main groups that received upstaging are shown in **bold**. p = 0.023 (Chi-Squared test).

**Table 5:** Comparison between the N clinical and pathological stages.

		T Classification (AJCC Cancer Staging System – 8^th^ edition)							
		pN0	pN1	pN2a	pN2b	pN2c	pN3a	pN3b	Total
T Classification (AJCC 8^th^ edition)	N0	37 (80.4%)	2 (4.3%)	1 (2.2%)	4 (8.7%)	1 (2.2%)	0 (0.0%)	1 (2.2%)	46 (57.5%)
	N1	8 (53.3%)	2 (13.3%)	2 (13.3%)	1 (6.7%)	1 (6.7%)	0 (0.0%)	1 (6.7%)	15 (18.8%)
	N2a	0 (0.0%)	1 (33.3%)	0 (0.0%)	0 (0.0%)	0 (0.0%)	0 (0.0%)	2 (66.7%)	3 (3.8%)
	N2b	1 (25.0%)	0 (0.0%)	0 (0.0%)	1 (25.0%)	0 (0.0%)	0 (0.0%)	2 (50.0%)	4 (5.0%)
	N2c	1 (33.3%)	0 (0.0%)	0 (0.0%)	1 (33.3%)	1 (33.3%)	0 (0.0%)	0 (0.0%)	3 (3.8%)
	N3a	0 (0.0%)	0 (0.0%)	0 (0.0%)	0 (0.0%)	0 (0.0%)	0 (0.0%)	0 (0.0%)	0 (0.0%)
	N3b	0 (0.0%)	0 (0.0%)	1 (11.1%)	2 (22.2%)	2 (22.2%)	0 (0.0%)	**4 (44.4%)**	9 (11.3%)
	Total	47 (58.8%)	5 (6.3%)	4 (5.0%)	9 (11.3%)	5 (6.3%)	0 (0.0%)	10 (12.5%)	80 (100.0%)

The main groups that received upstaging are shown in **bold**. p = 0.023 (Chi-Squared test).

## Discussion

Unilateral Vocal Fold Paralysis (UVFP) can cause significant laryngeal dysfunction, especially if the paralyzed fold is in an abducted position. Patients may present with disabling dysphonia, aspiration with risk of pneumonia, malnutrition, and vocal fatigue.^10^

Before planning the treatment for UVFP, it is relevant to assess the patient adequately to determine the etiology of the paralysis and identify the permanent or temporary causes and the specific characteristics of each patient that can influence the prognosis, such as clinical conditions and impact of dysfunction on quality of life.^11^

Initial treatment with speech therapy aims at compensating the paralyzed vocal fold, with an active vocal fold hyperfunction, and it can often be sufficient to relieve patient discomfort.

Surgery is indicated for cases in which the patient persists with quality-of-life impairment despite conservative treatment. It is recommended to wait approximately six months for a possible reversal of paralysis or satisfactory compensation from the opposite vocal fold. This period may be shorter if the paralysis is admittedly permanent, and the patient has life-threatening symptoms of aspiration.

Several techniques have been described, such as vocal fold injection with various substances, thyroplasty, and reinnervation procedures.

Type I (medialization) thyroplasty is currently the most commonly used treatment technique. However, it has limitations when a marked posterior glottic gap is present. In these cases, patients present severe dysphonia and frequent gagging. The use of an implant with posterior extension is aimed at solving this problem, but this is often not sufficient.

Zeitels et al.^7^, ^9^ and Zeitels^8^ proposed to associate adduction thyroplasty with arytenopexy and ipsilateral cricothyropexy. The technique consists of medialization and internal rotation of the arytenoid cartilage, which is fixed to the cricoid with suture. Next, type I thyroplasty is performed, in which the use of ePTFE is currently preferred. This material is easier to handle than silicone, and provides better titration of vocal fold medialization. In addition, it is available in most surgical centers because is used as material for vascular prostheses. The material volume required is smaller than that used when performing thyroplasty alone. This occurs because part of the medialization has already been achieved by the arytenopexy. Finally, the inferior cornu of the thyroid cartilage is attached to the medial portion of the cricoid. With this procedure, lengthening and increased tension are obtained in the paralyzed vocal fold.

The objective is a more efficient posterior glottic gap closure, in addition to obtaining a paralyzed vocal fold position in juxtaposition to the active vocal fold, tensioned and lengthened. With these modifications, functional results would be improved.

As a significant number of our patients presented marked posterior glottic gap with symptoms of aspiration and dysphonia, we began performing this technique at our otorhinolaryngology service in 2000.

This technique has not been widely used because of its greater complexity compared with simple thyroplasty and questioning about tolerability to the procedure under local anesthesia with sedation. There is also the fear that the greater surgical trauma would increase the risk of complication, especially of respiratory insufficiency with need of tracheostomy.

These fears have not been confirmed throughout our professional experience. All patients have tolerated the procedure under local anesthesia and sedation with performance of phonatory tasks during the surgery to assess the proper position of the vocal fold. No patient has presented respiratory distress per- or postoperatively, thus tracheostomy has not been needed. Patients were discharged on the first postoperative day. The functional results were considered satisfactory or optimal by both the patients and the attending physicians. Significant improvement in vocal quality and correction of aspiration events were observed. Patients did not present aspiration pneumonia, weight loss, or postoperative enteral dietary requirements. Adequate closure of the posterior glottic gap can be evidenced in postoperative laryngoscopy.

As late complications, two individuals presented endolaryngeal extrusion of the materials used for medialization. Despite their removal by direct laryngoscopy and general anesthesia, the functional results were not significantly affected. We believe that the vocal fold is maintained medialized not only because of the arytenopexy and cricothyropexy, but mainly because of the cicatrization process. Therefore, there was no need for surgical revision due to glottic dysfunction.

## Conclusion

Adduction arytenopexy with medialization thyroplasty and cricothyropexy can be performed under local anesthesia with sedation. This technique presents low degree of complication and excellent functional results.

Type 1 (medialization) thyroplasty presents the following advantages: better posterior glottic gap closure, more adequate placement of the paralyzed vocal fold in relation to the active vocal fold, and lengthening and tensioning of the paralyzed vocal fold. Therefore, the functional results are better regarding phonation, but mainly with respect to aspiration.

The disadvantage of this procedure lies in the fact that it is more complex, therefore more time consuming and with greater discomfort for the patient, being contraindicated in temporary paralysis due to its irreversible character.

## Funding

None.

## Declaration of competing interest

The authors declare no conflicts of interest.
